# The incidence of depression and anxiety disorders in spontaneous chronic urticaria patients

**DOI:** 10.5339/qmj.2023.sqac.16

**Published:** 2023-11-26

**Authors:** Anushree Bangera, Meghna Singh, Kiran Godse, Sharmila Patil

**Affiliations:** ^1^Department of Dermatology, Dr. D Y Patil Medical College, Navi Mumbai, India Email: anusalpa@gmail.com

**Keywords:** Chronic urticaria, anxiety, depression, HAMA, HDRS

## Abstract

**Objective**: A frequent condition known as chronic urticaria (CU) is characterized by the appearance of wheals, angioedema, or both. CU lowers the quality of life and may also result in psychological discomfort. The literature survey revealed few studies dealing with depression and anxiety in these patients. Hence, Hamilton scores for depression and anxiety were used in this study to evaluate the incidence of depression and anxiety in chronic urticaria patients.

**Methodology:** To evaluate CU patients’ levels of depression and anxiety, the Hamilton Rating Scale for Depression (HDRS) and the Hamilton Anxiety Rating Scale (HAMA) were applied. Moreover, in a control group of thirty healthy volunteers, thirty CU patients were included in this study. It was essential to observe the patients’ urticaria activity score, medications, age, gender, comorbidities, employment status, and income. When it came to levels of depression and anxiety, a comparison was made between the case group and the healthy group.

**Results:** In the CU patients’ group, the mean age was 26.9 years. The questionnaires showed that 14 (46%) subjects in the patient group had moderate to severe signs of anxiety, and 21 (70%) had moderate to severe symptoms of depression. Besides, in the control group,7 (23.3%) had moderate to severe signs of anxiety, and 8 (26.7%) had severe depression.

**Conclusion:** According to the study, individuals with CU exhibit depression and anxiety symptoms more frequently than the control group. Therefore, the possibility of mental comorbidities should be considered when treating individuals with CU.

